# Influence of Annealing Atmosphere on the Phosphatability of Ultra-High-Strength Automotive Steels

**DOI:** 10.3390/ma18133170

**Published:** 2025-07-04

**Authors:** Joongchul Park, Joonho Lee

**Affiliations:** 1 Department of Materials Science and Engineering, Korea University, 145 Anam-ro, Seongbuk-gu, Seoul 02841, Republic of Korea; heavymetal@posco.com; 2 Technical Research Laboratory, POSCO, 100, Songdogwahak-ro, Yeonsu-gu, Incheon 21985, Republic of Korea

**Keywords:** phosphatability, dew point, surface oxide, ultra-high-strength steel

## Abstract

This study investigates the effect of surface oxide control on the phosphatability of ultra-high-strength steel (UHSS) for automotive applications. Surface oxides were manipulated by adjusting the dew point to −50 °C and 0 °C during the annealing process, and the corresponding changes in phosphating behavior were examined. The surface characteristics of the samples were analyzed using X-ray photoelectron spectroscopy (XPS) and field-emission transmission electron microscopy (FE-TEM), while the phosphatability of the samples was evaluated through electrochemical measurements. The sample annealed at a dew point of −50 °C formed continuous Si and Mn oxide films (~10 nm), which significantly suppressed the phosphatability. In contrast, when annealed at 0 °C, internal oxidation occurred along the grain boundaries to a depth of about 3 μm, resulting in the formation of discontinuous Si and Mn oxides on the surface, which greatly enhanced phosphatability. This difference was also supported by OCP measurements: the −50 °C specimen showed a gradual increase in potential, whereas the 0 °C specimen rapidly reached −0.59 V and then stabilized. The findings of this study demonstrate that optimizing the annealing atmosphere provides an effective approach to enhance the phosphating performance of UHSS without the need for additional surface treatments.

## 1. Introduction

Recently, the demand for lightweight vehicles has been continuously increasing in the automotive industry due to the need for improved fuel efficiency and increasingly stringent emission regulations. Consequently, the application of ultra-high-strength steels (UHSSs), which can simultaneously achieve vehicle weight reduction and collision safety, has been expanding [1,2]. By utilizing the high strength of UHSS, it is possible to reduce the thickness of steel sheets compared to that of conventional steels, thereby achieving significant weight savings. In addition, enhancing the corrosion resistance of automotive steels has become increasingly important for extending vehicle service life and reducing carbon dioxide emissions from a life cycle assessment (LCA) perspective. To ensure the corrosion resistance of automotive steel, excellent paintability is essential, and this requires optimization of the phosphate treatment process, which serves as a critical pre-painting step [3,4]. If the phosphate coating does not grow uniformly on the steel surface, the coverage of the phosphate layer decreases, leading to reduced paint adhesion and, consequently, diminished corrosion resistance after painting. Automotive UHSS typically requires both high strength and excellent formability, which are achieved by alloying with elements such as Si and Mn. However, these alloying elements tend to diffuse to the surface during the annealing process, leading to selective oxidation that significantly affects the surface properties [5,6,7,8,9,10,11,12]. Zinc-based phosphate coatings are commonly employed as pre-painting treatments for automotive applications, and the effectiveness of this process is highly dependent on the nature of the surface oxides formed during annealing. In particular, the Si and Mn oxides formed on the surface of UHSS act as barriers between the steel substrate and the phosphate solution, thereby reducing the reactivity and overall quality of the phosphate coating compared to conventional steels. The quality of this phosphate coating is generally referred to as phosphatability [13].

The effects of surface oxides on phosphate coating have been extensively investigated with respect to various factors, including the Si/Mn ratio [14,15], the influence of oxide removal by pickling [16,17], Si content [18], surface decarburization [19], and the effect of boron [20]. Yan et al. [18] reported that increasing the Si content in low-carbon interstitial-free (IF) steel led to a deterioration in phosphatability due to the formation of selective oxides during heat treatment. Kim et al. [19] demonstrated that controlling the dew point during annealing of high-strength steel could suppress surface oxide formation and promote the development of C-lean ferrite, thereby improving phosphatability. Furuya et al. [20] found that the addition of B to Si and Mn containing high-strength steel resulted in the formation of B_2_O_3_, which induced structural changes in surface Mn_2_SiO_4_ and consequently reduced phosphatability. While previous studies have investigated the effects of alloy composition and annealing parameters on phosphatability, there remains a significant knowledge gap regarding the specific role of Si–Mn composite oxides. In particular, the formation mechanisms, chemical composition, and morphology of Si–Mn composite oxides under different annealing dew point conditions, and their direct correlation with phosphatability, have not been systematically elucidated. This gap is critical because Si–Mn composite oxides are frequently observed on the surface of ultra-high-strength steels, and their presence is known to strongly affect phosphate coating adhesion and uniformity.

Therefore, the objective of this study is to investigate the influence of dew point variation during annealing of UHSS on phosphatability and to propose potential improvement strategies through controlled dew point adjustment. This work builds upon our previous study, which examined the effect of dew point variation on the surface oxidation behavior of UHSS with a chemical composition of 0.2% C, 1.4% Si, and 2.35% Mn at dew points of −30 °C and +10 °C [21]. In the present study, the investigation was extended to a UHSS with a modified chemical composition (0.12% C, 1.0% Si) and a broader dew point range (−50 °C to 0 °C) to assess how variations in alloy chemistry and annealing atmosphere influence surface oxide characteristics and their subsequent effects on phosphatability. Surface oxides were characterized using FE-TEM and XPS, and the experimental results were further supported by thermodynamic calculations. The findings of this study are expected to provide valuable insights into optimizing annealing atmosphere conditions to enhance the phosphatability of UHSS in automotive applications.

## 2. Materials and Methods

The chemical compositions of the samples are listed in Table 1. The samples were initially hot-rolled to a thickness of 2.7 mm, followed by cold rolling to a final thickness of 1.4 mm. Heat treatment was conducted at 850 °C for 120 s in a 5% H_2_–N_2_ atmosphere using a hot-dip galvanizing simulator (HDG simulator, Iwatani, Munich, Germany). During annealing, the dew points were precisely controlled to −50 °C and 0 °C. The dew point was controlled by passing N_2_ gas through a bubbler to add water vapor to the gas. At this time, the temperature of the bubbler was controlled by a precision temperature controller included in the HDG simulator to adjust the saturated vapor pressure of the water vapor. The corresponding samples are hereafter referred to as DP-50 and DP0, respectively.

The annealed samples were sectioned into dimensions of 150 × 70 mm prior to the subsequent phosphating process. Initially, surface degreasing was performed by immersing the samples in an alkaline solution (FC-E2011, Nihon Parkerizing Co., Ltd, Tokyo, Japan) at 45 °C for 2 min to eliminate residual contaminants. The samples were then rinsed with water for 30 s, followed by surface conditioning in a zinc-based colloidal solution (PL-X, Nihon Parkerizing Co., Ltd, Tokyo, Japan) at 25 °C for 30 s. Phosphating was carried out by immersing the conditioned samples in a PALBOND L3065 (PB-L3065, Nihon Parkerizing Co., Ltd, Tokyo, Japan) bath at 35 °C for 120 s. After phosphating, the samples were washed with deionized water for 30 s and subsequently dried using high-pressure air.

The surface morphologies of both the annealed and phosphated samples, as well as cross-sectional features of the latter, were examined using field-emission scanning electron microscopy (FE-SEM). Quantitative depth profiles of elemental concentrations in the heat-treated samples were obtained via glow discharge spectroscopy (GDS). For detailed cross-sectional structural analysis, focused ion beam (FIB) milling was employed to prepare samples, which were then observed using an energy-dispersive field-emission transmission electron microscope (FE-TEM). Furthermore, cross-sectional elemental mapping analysis was conducted using a field-emission electron probe micro-analyzer (FE-EPMA). The chemical composition of surface oxides was characterized by X-ray photoelectron spectroscopy (XPS). XPS measurements were performed using a V.G. Scientific Escalab 250 spectrometer equipped with Al Kα radiation (CAE = 50 eV). For charge correction, the binding energy of the C 1s peak was set to 284.8 eV and used as a reference for all spectra. Phase stability of the surface oxides formed on the heat-treated samples was assessed using Factsage thermodynamic software (version 8.2). MnO, SiO_2_ MnSiO_3_, and Mn_2_SiO_4_ were calculated with the FToxid database, base metal phases with FSstel, and atmospheric gas with FactPS. The equilibrium oxygen partial pressure was determined at 850 °C under a 5% H_2_–N_2_ atmosphere (1 atm total pressure). The calculations assumed equilibrium conditions for the gas mixture and relevant oxide phases. To evaluate electrochemical reactivity, the samples were immersed in a phosphate solution, and the open circuit potential (OCP) was monitored at 35 °C for 3600 s using a Gamry Reference 600 potentiostat. Samples prepared by heat treatment under each dew point condition were evaluated independently three times each. OCP measurements were conducted by directly immersing degreased samples in the phosphate solution without any further surface pre-treatment, thereby enabling assessment of the inherent reactivity between the substrate and the solution.

## 3. Results and Discussion

### 3.1. Surface Structure and Composition

**Figure 1:**
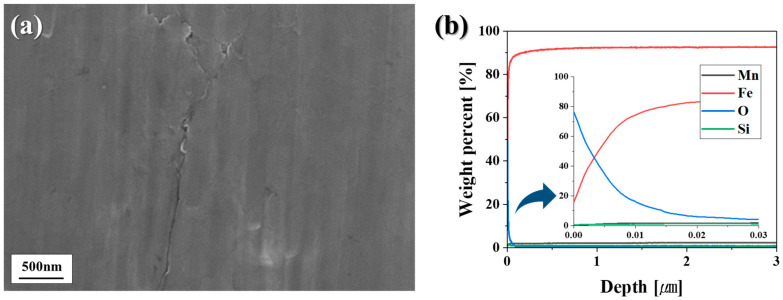
Surface analysis results of the sample before annealing: (**a**) FE-SEM images of surface oxide morphology; (**b**) GDS depth profiles.

Figure 1 presents the surface morphology and compositional analysis of the sample prior to annealing. As shown in Figure 1a, FE-SEM analysis indicates that selective oxide layers such as Si and Mn oxides are not formed on the steel surface before annealing. The depth profile analysis in Figure 1b further confirms that only Fe oxides with a thickness of less than 10 nm are present on the surface. Despite the addition of 1.0% Si and 2.35% Mn, as listed in Table 1, no Si or Mn oxides were detected on the sample surface prior to annealing. These results suggest that alloying elements such as Si and Mn diffuse toward the steel surface during annealing, thereby suppressing the formation of Si and Mn oxides before annealing.

**Figure 2:**
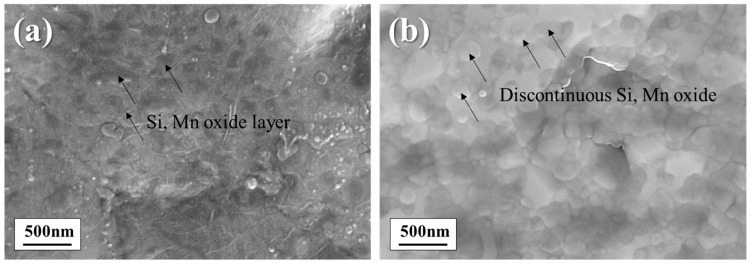
FE-SEM images of surface oxide morphology after annealing: (**a**) DP-50; (**b**) DP0.

Figure 2 presents the surface oxide morphologies of the annealed specimens prior to zinc phosphating. The DP-50 sample exhibits a continuous, film-like oxide layer on the surface (Figure 2a). Here, “film-like oxide” refers to oxides present on the surface in the form of a film. These continuous oxide films are expected to act as barriers and influence subsequent surface reactions such as zinc phosphating. In contrast, the DP0 sample (Figure 2b) displays a discontinuous, island-like distribution of surface oxides. These oxide islands are separated by exposed substrate areas, indicating incomplete surface coverage. In the exposed areas between these discontinuous oxides, sufficient phosphate crystal growth can occur through reactions with the phosphating solution.

**Figure 3:**
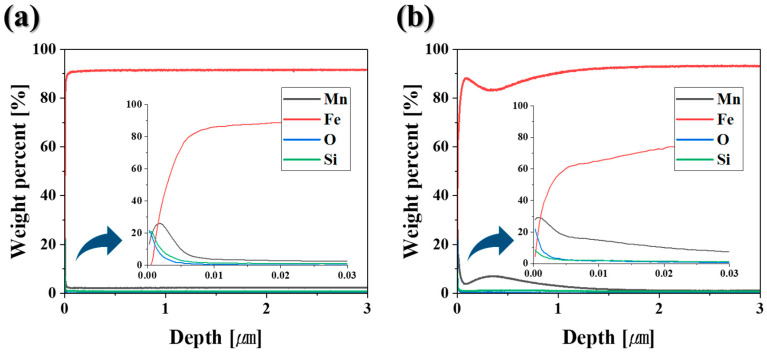
GDS depth profiles of (**a**) DP-50 and (**b**) DP0 samples after annealing.

Figure 3 displays the GDS depth profiling results for the DP-50 and DP0 samples. Concentration profiles of Fe, O, Si, and Mn were obtained to a depth of 3 μm, with the region within 30 nm from the surface shown in greater detail. In both samples, oxygen content decreases progressively with increasing depth; however, a higher dew point during annealing (from −50 °C to 0 °C) results in deeper oxygen penetration. The DP-50 sample exhibits significant enrichment of Si and Mn within approximately the top 10 nm of the surface. In contrast, while the surface Mn concentration in the DP0 sample is comparable to that of DP-50, the Si concentration is reduced to about half. Additionally, the Mn concentration in the DP0 sample decreases overall but shows a secondary increase at a depth of around 300 nm. This behavior is attributed to internal oxidation caused by the higher oxygen partial pressure at a dew point of 0 °C, meaning that oxygen diffuses into the alloy and causes sub-surface precipitation [5,8,9]. These variations in the surface Si and Mn ratios between the DP-50 and DP0 samples lead to differences in the types of oxides formed.

**Figure 4:**
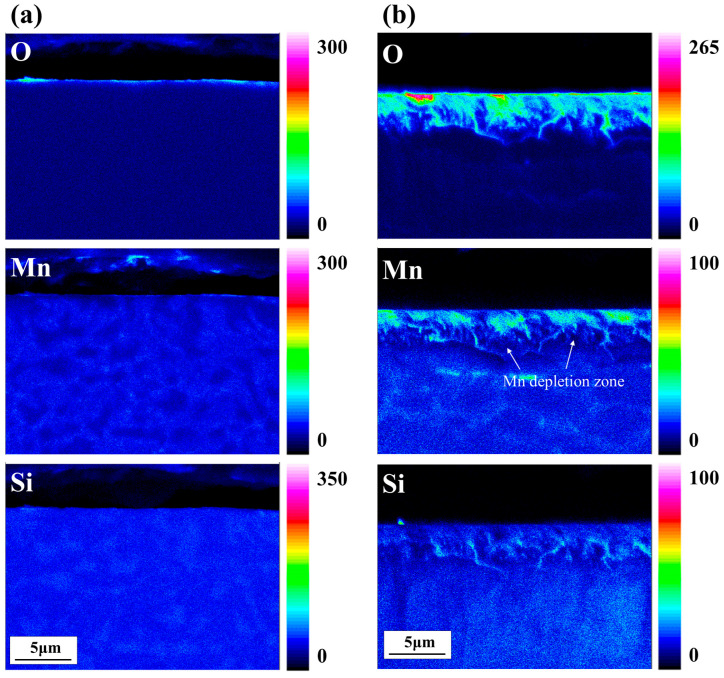
FE-EPMA elemental mapping of the samples after annealing: (**a**) DP-50; (**b**) DP0.

Figure 4 presents the EPMA elemental mapping results for the cross-sections of heat-treated specimens. In the DP-50 specimen (Figure 4a), a thin oxide film with a thickness of several nanometers is observed on the surface. In contrast, the DP0 specimen (Figure 4b) exhibits not only surface oxidation but also pronounced internal oxidation of Mn and Si, which is observed from the surface to a depth of 3–4 μm. The diffusion paths of these elements predominantly follow the grain boundaries, where deeper penetration of oxides is observed. Additionally, a distinct Mn-depleted zone is found adjacent to the grain boundary oxides.

It is well established that the diffusion of oxygen along grain boundaries is significantly faster than bulk diffusion, resulting in preferential oxidation and solute element migration along these boundaries during annealing [12]. Consequently, the formation of internal oxides is accompanied by the consumption of solute Mn, leading to the development of Mn-depleted zones around the oxidized grain boundaries. The thickness of the depleted region measured by EPMA is on the order of several hundred nanometers. Microstructural changes near the surface can significantly influence the mechanical properties of ultra-high-strength steels. Localized Mn depletion in this region may reduce strength and toughness, while the formation of internal oxides and related microstructural modifications can negatively affect ductility and crack resistance [22]. Therefore, accurate characterization of the depth and extent of solute depletion is crucial for optimizing heat treatment processes and achieving the desired properties in ultra-high-strength steels.

**Figure 5:**
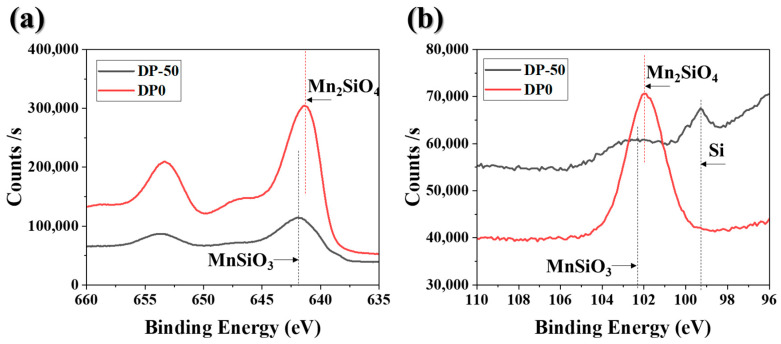
XPS spectra of for annealed samples: (**a**) Mn2p; (**b**) Si2p.

XPS analysis was conducted to determine the chemical composition of the surface oxides. Figure 5 presents (a) the Mn 2p spectrum and (b) the Si 2p spectrum. The fitting results are shown in Figure 5, where the main peaks are indicated by dashed lines. Reference binding energies for MnSiO_3_, Mn_2_SiO_4_, and bulk Si were taken from Grosvenor et al. [23] and other relevant literature [23,24], and the fitted peak positions were consistent with these references. In Figure 5a, the Mn 2p spectrum for the DP-50 sample shows a peak at 642.1 eV, which corresponds to MnSiO_3_, while the DP0 sample exhibits a peak at 641.7 eV, corresponding to Mn_2_SiO_4_. The binding energy difference between MnSiO_3_ and Mn_2_SiO_4_ is approximately 0.4 eV, which is consistent with previous reports [23]. In Figure 5b, the Si 2p spectrum for the DP-50 sample exhibits peaks at 101.9 eV for MnSiO_3_ and 99.2 eV for bulk Si. For the DP0 sample, Mn_2_SiO_4_ is the predominant oxide, with a peak observed at 102.3 eV. It was confirmed that the film-like MnSiO_3_ formed on the surface of the DP-50 sample reduced phosphatability. In contrast, Mn_2_SiO_4_ was present on the surface of the DP0 sample and did not significantly affect phosphatability.

### 3.2. Influence of Dew Point on the Formation of Phosphate Coating

**Figure 6:**
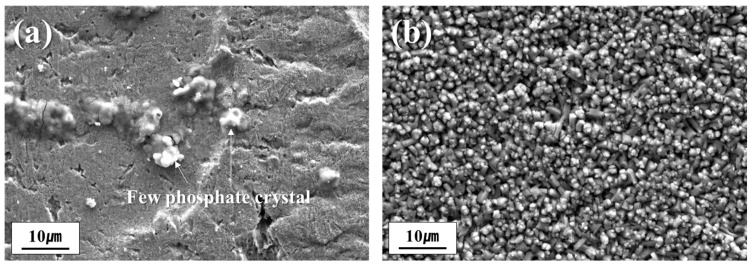
FE-SEM images of (**a**) DP-50 and (**b**) DP0 samples after phosphating.

Figure 6 illustrates the effect of dew point variation during annealing on the phosphatability of the samples. In the case of the DP-50 sample, phosphate crystal formation on the surface was minimal, resulting in most of the substrate remaining exposed, as shown in Figure 6a. Conversely, the DP0 sample exhibited a uniform distribution of fine phosphate crystals across the entire surface, as presented in Figure 6b. These results clearly show that the dew point during annealing plays a critical role in determining the surface condition of the steel, which in turn significantly affects the formation and morphology of phosphate coatings.

When steel is immersed in a phosphate solution, two sequential reactions—Fe dissolution and phosphate precipitation—take place, as illustrated in Equations (1)–(3) [25].

**Fe dissolution**

(1) $$Fe\left(s\right)+2H^{+}\to {Fe}^{2+}+H_{2}(g)$$

**Phosphate precipitation**

(2) $$3{Zn}^{2+}+2{H_{2}{PO}_{4}}^{-}+4H_{2}O\to {Zn}_{3}{\left({PO}_{4}\right)}_{2}\cdot 4H_{2}O(Hopeite)+{4H}^{+}$$

(3) $$2{Zn}^{2+}+2{H_{2}{PO}_{4}}^{-}+{Fe}^{2+}+4H_{2}O\to {Zn}_{2}Fe{\left({PO}_{4}\right)}_{2}\cdot 4H_{2}O(Phosphophyllite)+{4H}^{+}$$

Therefore, sufficient Fe dissolution at the steel surface is required for effective phosphate precipitation. However, stable oxides formed during annealing can hinder Fe dissolution, thereby reducing the phosphatability. The formation of these surface oxides is influenced by the annealing atmosphere. As shown in Figure 6, differences in phosphatability are observed when only the annealing atmosphere is varied, even for samples with identical compositions.

**Figure 7:**
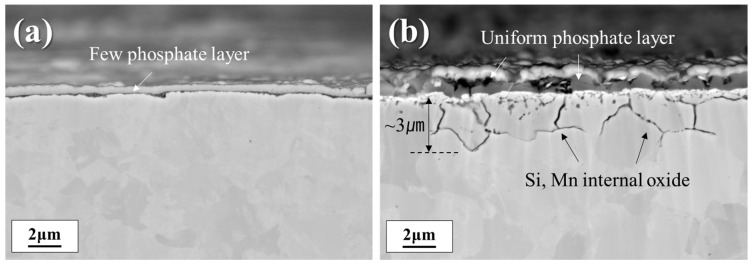
Cross-sectional FE-SEM images of samples after phosphating: (**a**) DP-50; (**b**) DP0.

To investigate the cross-sectional oxide distribution in samples annealed at different dew points, surface cross-sectional FE-SEM analysis was conducted on the phosphate-treated specimens, as shown in Figure 7. In Figure 7a, the DP-50 sample exhibits almost no surface phosphate layer, and a continuous, film-like oxide is present on the surface. In contrast, in Figure 7b, the DP0 sample displays a uniform phosphate layer on the surface, with evidence of internal oxidation extending to a depth of approximately 3 μm.

**Figure 8:**
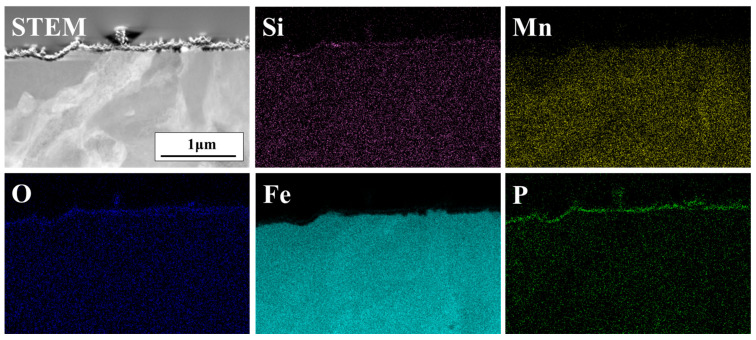
Cross-sectional FE-TEM images and EDS elemental mapping of DP-50 sample after phosphating.

Figure 8 shows the cross-sectional FE-TEM elemental mapping of the phosphate-treated DP-50 sample. A thin, film-like phosphate layer is distributed on the sample surface, indicating a small amount of phosphate treatment. Directly beneath the phosphate layer, a film-like Si-rich oxide is observed. As shown in the GDS depth profile analysis results in Figure 3, the thickness of this oxide is about 10 nm, and internal oxidation of the grain boundaries was not observed.

**Figure 9:**
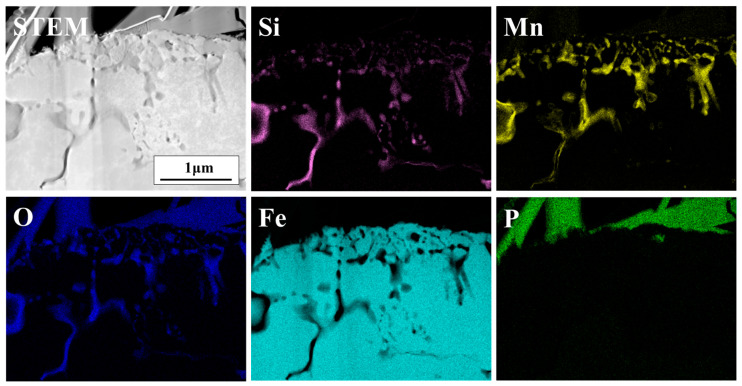
Cross-sectional FE-TEM images and EDS elemental mapping of DP0 sample after phosphating.

Figure 9 presents the cross-sectional FE-TEM mapping analysis of the sample annealed at a dew point of 0 °C. In Figure 9, discontinuous Si and Mn oxides are observed in the surface layer, and both Si and Mn oxides are distributed along the grain boundaries in the subsurface region to a depth of approximately 3 μm. Mn oxides are primarily distributed within the surface layer to a depth of about 2 μm, while Si-based oxides are mainly found in the innermost region of the internal oxidation zone. Internal oxidation tends to form oxides at grain boundaries, as oxygen diffusion along grain boundaries is much faster than bulk diffusion. Furthermore, as oxygen is consumed during the formation of surface oxides, the oxygen concentration gradually decreases with increasing depth. As a result, Mn-based oxides are formed near the surface, whereas Si-based oxides are formed in the subsurface layer.

Gong et al. [6] systematically investigated the internal oxidation behavior of high-strength steel under annealing conditions at 870 °C in a 10% H_2_–N_2_ atmosphere with a controlled dew point of +3 °C. They reported the formation of two distinct oxidation zones: MnO and x(MnO)·SiO_2_ phases were observed up to 1 μm from the surface, while Si-rich oxides were predominantly formed in the subsurface region, extending to a depth of approximately 4 μm. This distribution of oxides is generally consistent with the results presented in Figure 9 of the present study. However, a notable difference exists in the depth of internal oxide formation. These findings suggest that the depth and characteristics of internal oxidation are strongly influenced by variables such as heat treatment temperature, annealing time, and alloy composition.

To investigate the relationship between the dew point in the annealing furnace and the surface oxide formation behavior of steel, the oxygen partial pressure corresponding to different dew points was calculated. Specifically, Equations (4) and (5) were employed to determine the oxygen partial pressure for dew points of −50 °C and 0 °C in a 5% H_2_-N_2_ atmosphere, as described in reference [26]. In Equation (4), DP denotes the dew point, and in Equation (5), T represents the absolute temperature in Kelvin (K). The resulting values for water vapor and oxygen partial pressures at the experimental temperature of 850 °C are summarized in Table 2.

(4) $${log}_{10}{\left(p_{H_{2}O}\right)}_{sat.}=9.80\frac{DP}{273.8+DP}-2.22\;(\mathrm{If}\;DP\;\leq \;0\;{}^{\circ}C)$$

(5) $$\frac{1}{2}{log}_{10}p_{O_{2}}=3.00-\frac{13,088}{T}+{log}_{10}\left(\frac{{\left(p_{H_{2}O}\right)}_{sat.}}{p_{H_{2}}}\right)$$

**Figure 10:**
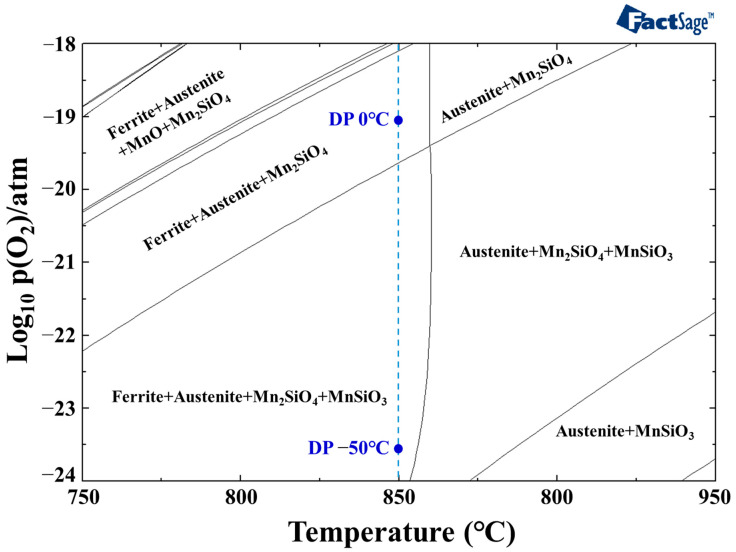
Phase stability diagram of the samples as a function of temperature and oxygen partial pressure.

To evaluate the influence of oxygen partial pressure—corresponding to the dew points listed in Table 2—on surface oxide formation during annealing, phase stability calculations were performed using FactSage thermodynamic software. The calculated phase stability for the experimental conditions is presented in Figure 10. At 850 °C, which corresponds to the experimental manufacturing conditions, Mn_2_SiO_4_ is stable on the sample surface when the dew point is 0 °C (p(O_2_) = 7.181 × 10^−20^ atm). However, the phase stability changes as the oxygen partial pressure decreases. As the dew point drops, Mn_2_SiO_4_ remains stable and MnSiO_3_ begins to form. Even at a dew point of −50 °C (p(O_2_) = 3.001 × 10^−24^ atm), both Mn_2_SiO_4_ and MnSiO_3_ are thermodynamically stable.

The presence of Si and Mn oxides formed during annealing impedes the phosphating process by acting as a barrier to the reaction between the steel substrate and the phosphating solution [14]. In particular, the control of SiO_2_ and MnSiO_3_ is critical, as these oxides tend to form continuous films on the steel surface [21]. Thermodynamic calculations indicate that, for the alloy system investigated in this study, annealing at 850 °C requires a dew point of at least −7 °C to suppress the formation of MnSiO_3_. If only Mn_2_SiO_4_ is present on the steel surface after annealing, the resulting discontinuous oxide layer can facilitate Fe dissolution during phosphating, thereby enhancing the reactivity of the steel in the phosphating process.

### 3.3. Electrochemical Behavior

**Figure 11:**
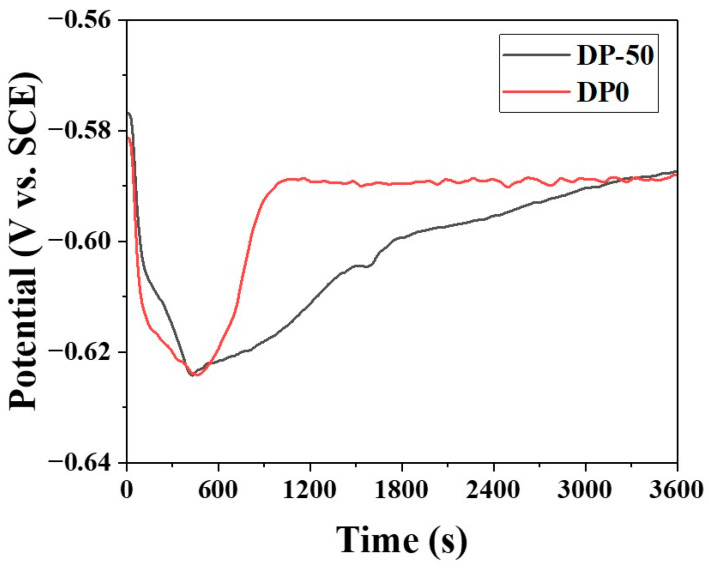
Results of OCP measurements of DP-50 and DP0 samples.

Figure 11 presents the open circuit potential (OCP) measurement results in phosphate solutions, which were conducted to investigate the influence of surface oxides on the initial phosphating reaction. Upon immersion, both samples exhibited a rapid decrease in potential, reaching their minimum values within the first 400–600 s. Specifically, the DP0 sample showed an initial potential of approximately −0.581 V, which decreased to a minimum of about −0.625 V at around 400 s. After reaching this minimum, the potential of DP0 increased rapidly and stabilized at approximately −0.590 V after 800 s, maintaining this value with minimal fluctuation until the end of the measurement. In contrast, the DP-50 sample also exhibited an initial drop in potential, reaching a minimum of about −0.625 V at around 400 s. However, the recovery of potential for DP-50 was slower compared to that for DP0, with the potential gradually increasing and stabilizing at approximately −0.59 V only after 3000 s. At the end of the measurement (3600 s), both samples converged to a similar potential value of about −0.590 V.

The phosphate treatment of steel plates typically involves a sequential process comprising an electrochemical attack, subsequent phosphate precipitation, and crystal growth [27,28]. Jiang et al. investigated the removal of surface oxides from 35CrMnSi steel using emery paper and monitored the open circuit potential (OCP) over time in phosphate solutions [28]. They observed an initial decrease in potential attributed to the electrochemical attack, followed by a gradual increase associated with the growth of phosphate crystals. These trends are consistent with the OCP results shown in Figure 11 of the present study. However, in this work, the phosphate reaction was conducted in the presence of Si and Mn surface oxides. The existence of these oxides acted as a barrier, generally retarding the initial reaction rate. Notably, in the DP0 sample, which possessed a film-type MnSiO_3_ oxide layer, the growth of phosphate crystals proceeded more slowly compared to that in the DP-50 sample.

**Figure 12:**
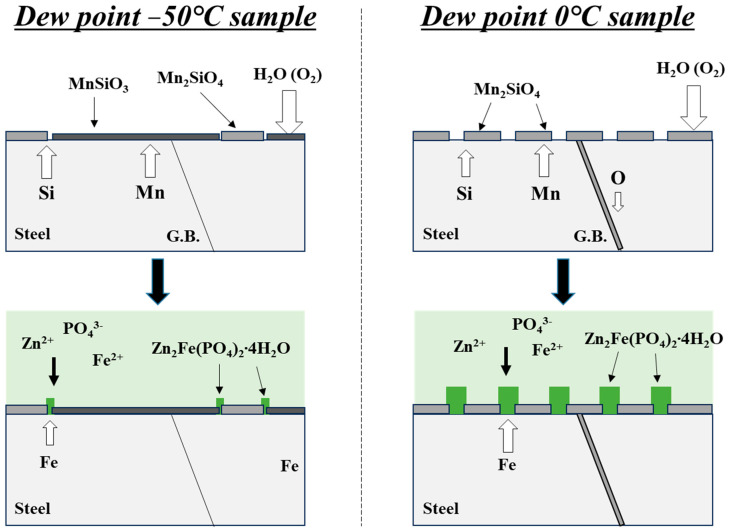
Schematic illustration of the surface oxide formation and phosphating reaction.

Figure 12 presents a schematic illustration of oxide formation and the subsequent phosphating reactions under two distinct dew point conditions. In the case of the DP-50 sample, the low oxygen partial pressure during annealing predominantly leads to the formation of Mn_2_SiO_4_ and MnSiO_3_ phases on the surface. Upon exposure to the phosphating solution, the MnSiO_3_ regions exhibit limited reactivity, while initial phosphate nucleation is observed locally in the vicinity of Mn_2_SiO_4_. Nevertheless, the overall growth of phosphate crystals remains insufficient within the reaction period, primarily due to the limited Fe dissolution. Conversely, the DP0 sample, subjected to a higher oxygen partial pressure, experiences internal oxidation, resulting in the formation of Mn_2_SiO_4_ along grain boundaries to a depth of approximately 3 μm from the surface. Additionally, discontinuous Mn_2_SiO_4_ particles are distributed on the surface. During the phosphating process, active Fe dissolution occurs at the exposed steel areas between these discontinuous Mn_2_SiO_4_, facilitating uniform phosphate nucleation and growth across the entire surface.

Many previous studies have investigated the influence of surface oxides on the hot-dip galvanizability of advanced high-strength steels for automotive applications, with most prior work focusing on optimizing surface conditions to improve zinc wettability and coating uniformity during the hot-dip galvanizing process [7,8,9,11,29,30,31]. In contrast, the present study systematically analyzed the direct effects of dew point variation during annealing on the characteristics and morphology of surface oxides and, consequently, on the phosphate treatability of ultra-high-strength steel. Notably, the results demonstrate that the formation of discrete, island-like Mn_2_SiO_4_ oxides at higher dew points enhances phosphatability, whereas the presence of continuous MnSiO_3_ films at lower dew points acts as a barrier to phosphate coating. This observation is particularly significant in comparison to hot-dip galvanizing, where continuous Mn–Si oxides are also known to hinder zinc adhesion and coating quality. Therefore, these findings not only provide a deeper understanding of the relationship between annealing atmosphere and phosphatability but also highlight the analogous mechanisms by which surface oxide control can simultaneously benefit both phosphate treatment and hot-dip galvanizing processes. This comparative analysis clearly demonstrates the advancements of the present study over previous related works.

## 4. Conclusions

In this work, the influence of dew point variation during annealing on the phosphate treatability of UHSS was systematically examined using microstructural characterization techniques. It was found that elevating the dew point from −50 °C to 0 °C during annealing led to a marked enhancement in phosphatability. This improvement is attributed to the alteration in oxygen partial pressure associated with the dew point change, which in turn modified the surface oxidation behavior of the steel. Under annealing conditions of 850 °C in a 5% H_2_-N_2_ atmosphere, a continuous, film-like MnSiO_3_ layer predominantly formed on the surface of the DP-30 specimen. Conversely, increasing the dew point to 10 °C promoted both external and internal oxidation, resulting in a transformation of the surface oxide composition from MnSiO_3_ to Mn_2_SiO_4_. The MnSiO_3_ layer present on the DP-50 sample surface acted as a barrier, impeding the phosphate reaction and thus diminishing phosphatability. In contrast, the Mn_2_SiO_4_ observed on the DP0 sample was distributed in a discrete, island-like morphology, which did not hinder the phosphating process. These findings demonstrate that raising the dew point during annealing can effectively enhance the phosphating response of ultra-high-strength steel. From an industrial perspective, the results of this study suggest that precise control of the dew point during annealing can serve as a practical and cost-effective strategy to improve the surface treatability of ultra-high-strength steels for automotive applications. The implementation of optimized annealing processes can enhance the adhesion and durability of coatings, thereby extending the service life of automotive components without the need for additional surface oxide removal steps. This is particularly important in terms of cost reduction and the development of environmentally friendly manufacturing methods. Future research should further investigate the effects of various alloying elements and their interactions with annealing conditions on the formation of surface oxides and steel microstructures, as well as the impact of microstructural changes caused by internal oxidation on the mechanical properties of steel. Such studies would provide deeper insights into the optimization of both chemical composition and processing parameters to achieve superior phosphatability.

## Figures and Tables

**Table 1:** Chemical composition of samples.

Sample	Chemical Analysis Results (wt pct)					Annealing Condition
	C	Si	Mn	P	Al	
DP-50	0.12	1.0	2.35	0.01	0.03	Dew point −50 °C
DP0	0.12	1.0	2.35	0.01	0.03	Dew point 0 °C

**Table 2:** Water vapor and oxygen partial pressures at dew points of −50 and 0 °C in a 5%H_2_-N_2_ atmosphere at 850 °C.

	DP 0 °C	DP −50 °C
$p_{H_{2}O}$(atm)	6.026 × 10^−3^	3.895 × 10^−5^
$p_{O_{2}}$(atm)	7.181 × 10^−20^	3.001 × 10^−24^

## Data Availability

The original contributions presented in this study are included in the article. Further inquiries can be directed to the corresponding author.
